# Impact of treatment for bacterial vaginosis on prematurity among Brazilian pregnant women: a retrospective cohort study

**DOI:** 10.1590/S1516-31802005000300004

**Published:** 2005-05-02

**Authors:** Rodrigo Pauperio Soares de Camargo, José Antonio Simões, José Guilherme Cecatti, Valéria Moraes Nader Alves, Sebastian Faro

**Keywords:** Bacterial vaginosis, Pregnancy, Premature infant, Perinatal care, Pregnancy complications, Vaginose bacteriana, Gravidez, Prematuro, Assistência perinatal, Complicações na gravidez

## Abstract

**CONTEXT AND OBJECTIVE:**

Bacterial vaginosis has been associated with prematurity and other perinatal complications. However, the efficacy of the treatment for preventing such complications has not yet been well established. The objective of this study was to evaluate the impact of treatment for bacterial vaginosis on a low-risk population of Brazilian pregnant women, in order to prevent prematurity and other perinatal complications.

**DESIGN AND SETTING:**

Observational retrospective cohort study, at the Obstetric and Gynecology Department, Universidade Estadual de Campinas (Unicamp).

**METHODS:**

Vaginal bacterioscopy results from 785 low-risk pregnant women were studied. Three different groups of women were identified: 580 without bacterial vaginosis during pregnancy, 134 with bacterial vaginosis treated using imidazoles (metronidazole, tinidazole, or secnidazole) during pregnancy, and 71 with bacterial vaginosis not treated during pregnancy. The diagnosis of bacterial vaginosis was based on Nugent's criteria, from the vaginal bacterioscopy performed during the first prenatal care visit.

**RESULTS:**

The frequency of prematurity was 5.5% among the women without bacterial vaginosis, 22.5% among those with untreated bacterial vaginosis and 3.7% among those with treated bacterial vaginosis. The risk ratios for perinatal complications were significantly higher in the group with untreated bacterial vaginosis: premature rupture of membranes, 7.5 (95% CI: 1.9-34.9); preterm labor, 3.4 (95% CI: 1.4-8.1); preterm birth, 6.0 (95% CI: 1.9-19.7); and low birth weight, 4.2 (95% CI: 1.2-14.3).

**CONCLUSION:**

The treatment of bacterial vaginosis significantly reduced the rates of prematurity and other perinatal complications among these low-risk Brazilian pregnant women, regardless of the history of previous preterm delivery.

## RESUMO

**CONTEXTO E OBJETIVO:**

A vaginose bacteriana vem sendo apontada como fator de risco para prematuridade e outras complicações perinatais. Entretanto, a eficácia do seu tratamento na prevenção destas complicações ainda não está esclarecida. O objetivo deste estudo foi avaliar o impacto do tratamento da vaginose bacteriana durante o pré-natal de baixo risco para a prevenção de prematuridade e outras complicações perinatais.

**TIPO DE ESTUDO E LOCAL:**

Coorte retrospectivo observacional, no Departamento de Tocoginecologia, Universidade Estadual de Campinas (Unicamp).

**MÉTODOS:**

Foram estudadas 785 gestantes de baixo risco com resultado da bacterioscopia de secreção vaginal. Foram identificados três grupos de mulheres: 580 sem vaginose bacteriana durante a gestação, 134 com vaginose bacteriana tratada com imidazólicos (metronidazol, tinidazol, ou secnidazol) durante a gestação, e 71 com vaginose bacteriana não tratada durante a gestação. O diagnóstico de vaginose bacteriana foi realizado utilizando os critérios de Nugent na bacterioscopia vaginal da primeira consulta.

**RESULTADOS:**

O parto prematuro ocorreu em 5,5% do grupo de mulheres sem vaginose bacteriana, 22,5% do grupo com vaginose bacteriana não tratada, e 3,7% do grupo com vaginose bacteriana tratada. A razão de risco para as complicações perinatais no grupo com vaginose bacteriana não tratada durante a gestação foi: 7,5 (intervalo de confiança, IC, de 95%: 1,9-34,9) para rotura prematura de membranas no pré-termo, 3,4 (IC de 95%: 1,4-8,1) para trabalho de parto prematuro, 6,0 (IC de 95%: 1,9-19,7) para parto prematuro e 4,2 (IC de 95%: 1,2-14,3) para baixo peso ao nascer.

**CONCLUSÃO:**

O tratamento da vaginose bacteriana reduziu significativamente os índices de prematuridade e outras complicações perinatais entre as gestantes de baixo risco, independentemente da história prévia de parto prematuro.

## INTRODUCTION

While a drastic reduction in neonatal mortality has been observed over recent years, especially in developed countries, the incidence of prematurity has remained practically constant. Although various programs and therapeutic regimens have been implemented to reduce the incidence of premature birth, the rate remains between 5% and 18%.^1^ These premature births account for 80% of all neonatal deaths.^1^ In the city of Campinas, State of Sao Paulo, Brazil, a survey indicated that 9.5% of newborns had low birth weight and 6.2% were premature.^2^

The multiple risk factors associated with prematurity include premature rupture of the membranes, previous premature birth, heavy physical work, black ethnicity and age greater than 30 years old.^3,4^ To prevent prematurity, the ideal course of action would be to predict which patients are at risk of premature delivery and then implement effective intervention.^5-7^

In several studies, bacterial vaginosis has been associated with prematurity and other adverse outcomes, independent of other known risk factors.^8-12^ While bacterial vaginosis has been associated with preterm delivery and low birth weight, randomized trials for the treatment of bacterial vaginosis during pregnancy have not always demonstrated a reduction in such outcomes.^11,13^

The treatment of choice for bacterial vaginosis has been metronidazole in the United States.^14^ Trials have found a signifi cant reduction in the rate of premature births among pregnant women who presented high risk of prematurity and whose bacterial vaginosis was treated with metronidazole.^15,16^ On the other hand, there are similar trials that did not find a decrease in the prematurity rates among low-risk populations.^13,17,18^ The lack of beneficial effect from metronidazole may, however, be related to the dosing regimens used.^18^

A recent meta-analysis that compared treatment of bacterial vaginosis with placebo found that the evidence did not support systematic screening for and treatment of bacterial vaginosis for all pregnant women, aimed at preventing premature birth, postpartum infection and neonatal sepsis.^11^ However, there was evidence that indicated that, for women with a history of premature birth, treatment of bacterial vaginosis could prevent subsequent preterm birth.^11^

A Brazilian study has found 20% prevalence of bacterial vaginosis among asymptomatic pregnant women.^12,19^ In addition, a significantly increased risk of neonatal complications was found among this population.^12^ The present study was conducted to evaluate the impact of treatment for bacterial vaginosis among a population of Brazilian pregnant women, in order to prevent prematurity and other undesired outcomes from pregnancy.

## METHODS

In an attempt to determine whether the treatment of bacterial vaginosis during prenatal care reduced the incidence of preterm delivery, we conducted the present observational retrospective cohort study among all 785 women who were attending the Obstetric Service at the Universidade Estadual de Campinas (Unicamp), Brazil, between January 1997 and March 1999. The study made use of the results from vaginal Gram stains available in the patients' records.

The women were categorized in three different groups that were identified with regard to bacterial vaginosis: one group without detection of bacterial vaginosis at the first prenatal care visit (580 women); one group with bacterial vaginosis treated with imidazolic antibiotics during pregnancy (134 women); and one group with bacterial vaginosis that was not treated during the gestation (71 women). Institutional Review Board approval was obtained for the study.

The diagnosis of bacterial vaginosis was based on Gram's stain, using Nugent's criteria.^20^ Vaginal swab samples had been collected from each pregnant woman during the first prenatal care visit. The decision regarding which antiprotozoal agent to use (metronidazole, tinidazole or secnidazole) was based on the attending physicians' choice and the availability of the agents at the clinic.

Women were not included in this study if they had: gestational age greater than 34 weeks at the first prenatal care visit, diabetes, arterial hypertension of any etiology, multiple gestations, polyhydramnios, placenta previa, abruptio placentae, fetal malformation, or infection of the genital tract by *Trichomonas vaginalis, Chlamydia trachomatis* or *Neisseria gonorrhoeae*. All these exclusion criteria were based on the appearance of such diagnoses in the patient's chart.

Tinidazole and secnidazole have been approved for use in Brazil, and also in Europe, and are comparable to metronidazole with regard to efficacy. Tinidazole and secnidazole are frequently used as an alternative to metronidazole, depending on their availability in the hospital pharmacy, and they are dispensed to patients at Unicamp without charge.

As there are essentially no known differences in the treatment regimens with regard to their efficacy for treating bacterial vaginosis at the time of the study, we found that several therapeutic regimens had been used: metronidazole, 750 mg/day, orally for seven days (57 women); metronidazole, tinidazole or secnidazole, 2 g orally, single dose (three, four and four women, respectively); or metronidazole or 0.75% tinidazole gel, one full applicator, intravaginally once a day for seven days (36 and nine women respectively). The imidazole administration route was not stated in the clinical record files for 21 patients with treated bacterial vaginosis.

The therapeutic regimen was chosen at the discretion of the attending clinician. They had greatly differing opinions regarding treatment. Since, at that time, treatment was not considered to be mandatory, several attending physicians did not treat cases of bacterial vaginosis. Furthermore, some women's clinical records confirmed that bacterial vaginosis had been treated using one of these drugs, but the imidazole administration route was not stated.

Preterm-premature rupture of membranes was defined as rupture of the amniotic membranes before the 37^th^ week of pregnancy and before the onset of labor.

Premature labor was defined as the occurrence of regular and persistent uterine contractions before the 37^th^week of pregnancy. In addition, the patient had to demonstrate cervical dilatation and effacement that required treatment using tocolytics.

A premature birth was defined as a newborn with less than 37 weeks (259 days) of gestation. The length of gestation was determined from the date of the last menstrual period, obstetric sonography, and/or examination of the newborn using the Capurro method.^21^

Newborns weighing less than 2,500 g were considered to have low birth weight.

Infants were considered to present neonatal morbidity if they developed complications during the newborn period requiring admission to the neonatal semi-intensive and/or intensive care unit (for example, due to respiratory distress, neonatal anoxia, hypoglycemia, or sepsis).

Maternal morbidity was considered to be present if there was the presence of endometritis and/or infection of a surgical wound that required hospital admission.

Chorioamnionitis was defined as maternal fever (temperature ≥ 38° C) and amniotic fluid with odor.

The statistical analysis was performed using the chi-squared and Fisher exact tests for category variables, and variance analysis for quantitative variables. For the main dependent variables, the risk ratio was calculated with a 95% confidence interval. Furthermore, multiple logistical regression analysis was performed to compare the groups with treated bacterial vaginosis and untreated bacterial vaginosis.

## RESULTS

The average ages in years were 26.6 (± 6.9), 24.8 (± 6.9) and 24.3 (± 6.1), respectively, for the groups without bacterial vaginosis, with treated bacterial vaginosis and with untreated bacterial vaginosis. Eighty-two percent of the samples (647/785) were collected before 30 weeks of gestation, and the mean gestational age at the time of collection was 23.5 weeks. There were no statistically significant differences between the three groups with regard to the sociodemographic characteristics, habits and obstetric antecedents, except for the smoking habit, which was higher in the groups with bacterial vaginosis.

There were no significant differences in preterm-premature rupture of amniotic membranes, premature labor, premature birth and low-birth-weight newborns between the group without bacterial vaginosis and the treated group. On the other hand, there was a statistically significant difference between the groups with treated and untreated bacterial vaginosis, with regard to adverse perinatal outcomes (Table 1).

**Table 1:** Comparison of adverse perinatal outcomes between pregnant women groups with treated and untreated bacterial vaginosis in a university hospital in Brazil

Adverse perinatal outcomes	Treated bacterial vaginosis		Untreated bacterial vaginosis		p-value
	n	%	n	%	
Preterm-premature rupture of amniotic membranes	3/134	2.2	12/71	16.9	< 0.001*
Premature labor	11/134	8.2	20/71	28.2	< 0.001†
Premature birth	5/134	3.7	16/71	22.5	< 0.001‡
Low birth weight	5/134	3.7	11/71	15.5	0.003§
Puerperal infection	–	–	4/71	5.6	0.013
Amnionitis	–	–	2/71	2.8	0.045
Length of stay in neonatal					
intensive care unit	1/134	0.7	4/71	5.6	0.049
≥ 2 days					
Neonatal morbidity	9/134	6.7	13/71	18.3	0.020

* *Risk Ratio (95% CI): 7.55 (1.90 - 34.93)*

† *3.43 (1.46 - 8.15)*

‡ *6.04 (1.97 - 19.76)*

§ *4.15 (1.27 - 14.35).*

The multiple logistical regression analysis for the treated and untreated bacterial vaginosis groups identified younger age and untreated bacterial vaginosis as factors that were associated with preterm-premature rupture of amniotic membranes. The factors associated with premature labor were preterm-premature rupture of amniotic membranes and untreated bacterial vaginosis, while the factors associated with premature birth were preterm-premature rupture of amniotic membranes, preterm labor and untreated bacterial vaginosis. Finally, the regression model only identified premature birth as a statistically significant factor associated with low birth weight.

There was no occurrence of puerperal infection or amnionitis in the group with treated bacterial vaginosis and low frequency of this in the group without bacterial vaginosis. Conversely, the occurrence of puerperal infection, amnionitis and neonatal morbidity, and the length of stay in neonatal intensive care units, was significantly higher in the group with untreated bacterial vaginosis than in the group with treated bacterial vaginosis (Table 1).

An analysis of the subgroups, excluding women with a history of previous preterm delivery, was also performed. No significant differences in the incidence of perinatal complications was found between the groups without bacterial vaginosis and with treated bacterial vaginosis, except with respect to preterm-premature rupture of amniotic membranes (0.2% versus 2.5%; p = 0.026).

On the other hand the incidence of most perinatal complications was significantly higher in the group with untreated bacterial vaginosis than in the group with treated bacterial vaginosis (Table 2).

The relationship between the incidence of perinatal complications and the different routes of treatment (oral or vaginal) was also analyzed. No significant differences were found with regard to preterm-premature rupture of membranes, premature labor and low birth weight in the groups with oral or vaginal treatment. Premature birth was significantly more frequent in the subgroup with vaginal treatment (p = 0.008).

## DISCUSSION

The results of this study have demonstrated that the treatment of bacterial vaginosis had a favorable impact on reducing the occurrence of preterm-premature rupture of amniotic membranes, premature labor and birth, low birth weight, and neonatal morbidity among Brazilian women. These complications were significantly less frequent among the pregnant women with treated bacterial vaginosis than among those with untreated bacterial vaginosis. Furthermore, the frequencies were similar between the women with treated bacterial vaginosis and the controls (those without bacterial vaginosis).

**Table 2:** Comparison of adverse perinatal outcomes between the pregnant women groups with treated and untreated bacterial vaginosis, excluding the women with a history of preterm delivery* in a university hospital in Brazil

Adverse perinatal outcomes	Treated bacterial vaginosis		Untreated bacterial vaginosis		p-value
	n	%	n	%	
Preterm-premature rupture of amniotic membranes	3/120	2.5	12/66	18.2	< 0.001
Premature labor	10/120	8.3	17/66	25.7	0.002
Premature birth	5/120	4.2	14/66	21.2	< 0.001
Low birth weight	5/120	4.2	9/66	13.6	0.037
Puerperal infection	–	–	4/66	6.1	0.015
Amnionitis	–	–	1/66	1.5	0.215
Neonatal morbidity	4/120	3.3	5/66	7.6	0.284

* *Fourteen women had previous preterm delivery among the women with treated bacterial vaginosis and five in the untreated bacterial vaginosis group.*

The three groups of women were homogeneous with regard to obstetric and sociodemographic characteristics. In general, these variables had homogeneous distribution in the sample, except for smoking. Even so, when smoking was compared only between the two groups with bacterial vaginosis (treated and untreated), the difference was not significant for this variable, either. Since these two groups did not differ in demographic characteristics, we can assume that these demographic characteristics had no impact or association with preterm birth in this study.

The women were not randomized and there were no criteria used in making the decision of whether to treat (and which route to use for the imidazole) or not to treat. The attending physician had the responsibility for making these decisions, and this may have introduced a significant selection bias.

Preterm labor was the most prevalent neonatal outcome for all groups. The occurrence of preterm labor was three times higher in the group with untreated bacterial vaginosis than among the treated women. Preterm-premature rupture of amniotic membranes occurred seven times more frequently in the group with untreated bacterial vaginosis than in the group with treated bacterial vaginosis. A recent publication has also demonstrated that treatment of bacterial vaginosis during the prenatal period resulted in a reduction in the rate of premature rupture of the membranes, but only for women at higher risk of prematurity, i.e. those with a history of preterm birth.^11^ This suggests that women at risk of preterm delivery should be screened, if possible prior to conception or early in the pregnancy, and treated. Women with a history of preterm delivery and bacterial vaginosis may have subclinical or asymptomatic endometritis and therefore may experience an inflammatory response when becoming pregnant. Developing recurrent episodes of bacterial vaginosis may induce an enhanced inflammatory response, thereby resulting in premature rupture of amniotic membranes and preterm labor, or preterm labor with intact amniotic membranes. Korn et al.^22^ showed the existence of asymptomatic endometritis in non-pregnant women.

The estimated risk of preterm birth was six times higher among women with untreated bacterial vaginosis than in the group with treated bacterial vaginosis. The high rate of preterm birth among women with untreated bacterial vaginosis is perhaps explained, in part, by the design limitations of the study. The relative risk of preterm birth among women with bacterial vaginosis ranges from 1.5 to 3.0 in most of the published studies.^8,9,23,24^ These rates are rather lower than the rate found in the present study. However, Hay et al.^25^ also found a relative risk of 7.3, which is very similar to our results. In addition, other published data have also suggested that treatment of bacterial vaginosis during pregnancy can reduce the risk of neonatal complications, especially prematurity.^3,16,22^

The incidence of low-birth-weight infants was four times higher in the group of women with untreated bacterial vaginosis than among those with treated bacterial vaginosis. Low birth weight has been assumed to be a result of the preterm-premature rupture of amniotic membranes and the preterm birth, rather than a direct complication of bacterial vaginosis.

Puerperal infection was a significant outcome in this study in the group with untreated bacterial vaginosis. In all cases of puerperal infection, the delivery was by means of cesarean section. It is important to highlight that all the women who underwent cesarean section received cephazolin 1 g, IV in a single dose, immediately after cutting the umbilical cord, as a routine prophylactic procedure of our service. It seems that this antibiotic prophylaxis is not as effective as expected in preventing puerperal infection among women with bacterial vaginosis at the time of delivery. In the literature, there is also evidence that bacterial vaginosis can be an important risk factor for endometritis after cesarean deliveries, in spite of antibiotic prophylaxis using cephalosporin.^26^

Comparing the oral and intravaginal treatment regimens, prematurity occurred more frequently among women treated with intravaginal antibiotic. There was a limitation on this evaluation, because 21 patients with treated bacterial vaginosis had no data available on their charts to show the route of imidazole administration. Also, the patients who were treated with antibiotics received one of six possible regimens. Such variance in treatment makes it difficult to draw conclusions about what actual effect there was. Nevertheless, this result is in agreement with a recent meta-analysis demonstrating that systemic treatment during pregnancy was more efficacious than intravaginal metronidazole.^11^

On the other hand, Carey et al.^13^ did not find a reduction in the rates of prematurity or other neonatal complications. However, they used 2 g of metronidazole twice for treatment of bacterial vaginosis. Treatment of bacterial vaginosis with a single dose of metronidazole is associated with higher rates of relapse than is treatment over a long period of time, especially when long-term cure rates are analyzed.^14^

Admission of the newborn to the neonatal intensive care unit was significantly more frequent for the group of women with untreated bacterial vaginosis. A reduction in neonatal intensive care unit admissions can significantly reduce costs. Müller et al.^27^ recently demonstrated the cost benefit of diagnosis and treatment of bacterial vaginosis during pregnancy for reducing admissions to the neonatal intensive care unit in Germany. This can have an even greater importance in developing countries.

However, the issue of screening for and treating bacterial vaginosis among pregnant women remains unsettled. The current literature suggests that only women with a history of preterm birth can benefit from treatment of bacterial vaginosis.^11^ In the present study, an analysis of women not at risk of preterm birth (i.e. with no history of previous prematurity) found that these women also benefited from treatment for bacterial vaginosis.

## CONCLUSION

This study shows that treating bacterial vaginosis during pregnancy could significantly reduce the risk of prematurity among Brazilian pregnant women. Treating bacterial vaginosis in these pregnant women resulted in a significant reduction in premature rupture of the membranes, preterm birth, postpartum endometritis, and neonatal sepsis. This positive effect was also demonstrated among pregnant women without a history of premature birth. However, because the currently available knowledge is still controversial, and this study had some limitations, similar studies are needed in distinct populations, before universal screening and treatment of bacterial vaginosis among pregnant women in developing countries can be recommended.
